# Do Theory of Mind and Mental Time Travel abilities build on joint cognitive foundations?

**DOI:** 10.1098/rsos.241960

**Published:** 2025-06-11

**Authors:** Lydia Paulin Schidelko, Leonie Baumann, Marina Proft, Hannes Rakoczy

**Affiliations:** ^1^ Department of Cognitive Developmental Psychology, Georg-August-Universität Göttingen, Göttingen, Niedersachsen, Germany

**Keywords:** Theory of Mind, Mental Time Travel, meta-representation, recursion, possibility reasoning, counterfactual reasoning

## Abstract

Higher cognition is special in that it goes beyond representing the here and now. Two relevant forms of such thinking are *Theory of Mind* (ToM) that enables us to represent others’ perspectives, and *Mental Time Travel* (MTT) that enables us to represent other points in time. The present studies investigate how these capacities are related in development. Do they build on the same cognitive foundations and thus emerge together? Do higher-order forms of the two abilities rely on analogous recursive embedding and thus progress in parallel and coordinated ways? We addressed these questions in four studies with 3- to 9-year-old children (*N* = 395). ToM was operationalized as first-, second- and third-order false belief understanding. MTT was operationalized as reasoning about future possibilities (first-order), counterfactual reasoning (second-order) and anticipating counterfactual emotions (third-order). Study 1 shows a stepwise development of both ToM and MTT and a moderate consistency of performance patterns. However, across all four studies, we did not find robust correlations between first-, second- and third-order tasks of ToM and MTT, respectively. Overall, these results show stepwise and parallel trajectories in ToM and MTT, but do not provide stringent evidence for a joint cognitive foundation of the two capacities.

## 1. Introduction

One hallmark of higher cognition is that it goes beyond representing the here and now. We do not only represent the world from our own perspective, but also how it appears to other agents—what they see, believe and desire (*Theory of Mind*). Moreover, we do not only think about how the world is now, but also imagine other points in time—how the world was, could be or will be (*Mental Time Travel*). The present paper examines how these two ways of going beyond representations of the here and now are related in development. Do they share a joint cognitive foundation? Do they emerge together? And do they progress developmentally in parallel and coordinated ways?

Theory of Mind (ToM) is the social–cognitive ability to ascribe and reason about mental states [1]. At the conceptual heart of ToM lies meta-representation: the capacity to represent how others represent the world even when these representations diverge from the interpreter’s own perspective and from reality. ToM emerges in the course of a major conceptual transition at around age 4 [2,3]. Around this time, children come to master the litmus test for ToM, the so-called false belief task (in which they need to track a protagonist’s subjective and outdated belief) as well as many other conceptually related tasks that differ with regard to topic, format and surface structure but all require perspective taking (for an overview, see [3]).

Mental Time Travel (MTT) is the ability to think and reason about different points in time, such as the past (episodic memory), the future (foresight) and how they relate to the present [4–10]. Developmental research on the emergence of future-oriented forms of MTT has suggested that the ability to reason about future possibilities emerges in the course of a major conceptual transition at around age 4 [7,11,12]. In prototypical tasks, participants are faced with a situation that presents them with multiple, still open future possibilities. Crucially, the possibilities are mutually exclusive, so only one of them will be realized. This requires the participant to represent *that* as well as *how* these future possibilities relate to the same present state of uncertainty.

For instance, in a task designed by Beck and colleagues, children are asked to put out mats to catch a mouse that could come out of either one of two exits of a forked (inverted y-shaped) slide. The results show that only children aged 5 and older succeed in preparing for the uncertain future event by putting one mat under each exit of the forked slide [13]. A simplified version of this task [7], in which participants cover the exits of a forked tube with their hands, found success in slightly younger children at age 4. In yet another version of this task, two objects come down two slides simultaneously: one slide is non-branching with one exit, the other forks into two exits. Participants are asked to place a wagon under one of the exits to catch one of the two objects. In this set-up, children have to reflect on all options simultaneously and compare them with each other. Children aged 5 and older succeed by choosing the certain option (i.e. placing the wagon under the non-branching slide) over the uncertain options (i.e. placing the wagon under the forked slide) [14,15]. Other studies that follow related task structures have led to similar results [16].

Children thus start to demonstrate first-order MTT (reasoning about future possibilities) from around age 4 to 5 (e.g. [13,14,16,17]). Similarly, children reliably come to solve false belief and conceptually related tasks that require meta-representational ToM at the age of 4 [18]. Are these parallel developmental trajectories a mere coincidence or could they reflect deep and underlying cognitive commonalities in the form of a joint (meta-)representational foundation [19]? One possibility is that both ToM and MTT may build on the same (neuro-)cognitive capacity for simulation and projection. For one, explicit ToM requires simulation to shift one’s perception from the immediate environment to alternative perspectives. In a similar sense, imagining future possibilities requires projection to shift one’s perception from the immediate environment to alternative, imagined future events [6,8,20].

Another, not necessarily mutually exclusive, possibility is that both ToM and MTT do not only build on overlapping processes of projection and simulation, but that they are based on the same underlying cognitive ability to hold meta-representations [7,19,21–23]. Generally, the ability to hold meta-representations enables us to represent the relation between the actual state of reality and mere representations of reality. In the case of explicit ToM, it is obvious that meta-representation in some sense underlies the capacity to represent the subjective perspectives (i.e. representations) of agents [2,24,25]. It is prima facie somewhat less obvious in which ways MTT may build on meta-representation. Different arguments have been made to this effect. One is that MTT requires a particular form of autonoetic representation of one’s own past or potential future experiences and perspectives, and thus a form of meta-representation [23] (see also [26,27]). Another argument is that MTT requires a meta-representational grasp of the way temporal representations relate to the actual state of reality [4,7,21]. For example, remembering that *p* and predicting that *p* involve specific representational relations of the content entertained (*p*) to present or future states of the world. In the forked slide task, for instance, the ability to hold meta-representations allows one to realize that a prediction of a future outcome (e.g. that the object will come out of the left exit) is merely a representation of a potential future reality and is not necessarily how the future will unfold. Put another way, meta-representation enables us to represent that any given representation of the future might be false, just as it enables us to represent that any given representation of reality from another mind might be false [7,17]. Overall, holding meta-representations allows us to relate mere representations of reality to the actual state of reality by embedding these representations within a specific representational context—be that a different point in time or the mind of another [21,25].

Against this background, the first research question of the present study is whether the two ways of going beyond representations of the here and now under consideration—ToM and MTT—build on some form of joint cognitive foundation and therefore emerge and develop in parallel and coordinated ways [19,22]. One potential indirect indicator for this joint cognitive foundation would be (co-)emergence in the same age window. However, co-emergence by itself is not a sufficient indicator of joint foundations. More direct evidence from correlational studies would be needed, such that when children acquire one capacity, they also acquire the other. As reviewed above, evidence from studies that investigated the two abilities separately shows that they do seem to emerge roughly around the same age (e.g. [13–18]). Moreover, a recent study that tested both abilities in children (reasoning about future possibilities and false belief reasoning) found no difference in the age of onset [28]. However, beyond this, hardly any studies have tested more stringently for developmental correlations. One recent study did so but failed to find evidence for correlations between ToM and episodic memory or episodic future thinking [29]. The present study thus aims to investigate the co-emergence and developmental correlation of ToM and MTT more stringently and systematically.

If indeed ToM and MTT rest on the same meta-representational foundation, the next question would be whether this only reveals itself in the joint *emergence* of the two capacities (i.e. first-order ToM and MTT), or whether subsequent more complex, higher-order forms of ToM and MTT, respectively, are also developmentally related [19,22]. In both ToM and MTT, more complex and higher-order forms of reasoning emerge by recursive embedding. In ToM, meta-representation can be theoretically iterated ad infinitum (‘A thinks that B thinks that C thinks…that *p*’). Children ascribe first-order beliefs at around age 4, and attribute second-order mental states from around age 5 to 6 (‘A thinks that B believes that…’) [30]. Very little is known about children’s development of higher-order ToM beyond the second order of recursion. First evidence shows that children become able to attribute third- and fourth-order mental states only during middle childhood from around age 8 to 10 [31,32].

Similarly, recursive iteration can generate more complex and higher-order forms of MTT in which additional levels of temporal representations can be represented and embedded within each other: for instance, remembering past moments in which one thought about the more distant past, or imagining that in the future one will make plans for the more distant future. Recursive temporal embedding can also involve switching back and forth between future and past perspectives, such as remembering moments in the past in which one thought about the future or imagining that in the future one will look back on the relative past [7].

In the framework devised by Gautam *et al.* [19], one example of first-order MTT is the ability to reason about future possibilities, which children seem to acquire from around age 4. One example of second-order recursive MTT is counterfactual reasoning about past and present events (as in ‘What would the US look like today if Trump had won the 2020 election?’). When reasoning counterfactually, one has to mentally go back in time to imagine how an alternative past (level 1) would have affected the relative future and would thus have led to an alternative present (level 2). Crucially, the relation between multiple simultaneous representations of the world—reality and counterfactual alternative—must be represented in this process [33–36], specifically, as alternative versions of the very same moment in time that originate from a common past [7,37]. Stringent tests for counterfactual reasoning involve cases of overdetermination: events A and B both independently produced effect E. What if A had not happened? To give the correct answer (E would still have happened, caused by B), children cannot take simpler shortcuts but have to engage in true counterfactual reasoning [36] (e.g. [38,39]). In a task that used physically caused events, children were able to answer counterfactual test questions about doubly determined scenarios correctly at age 6 [35] (but see also [40] who show this ability already in children at the age of 4). Another example of second-order recursive MTT is the experience of counterfactual emotions such as regret and relief, which emerges around the same time [19,41–47]. These emotions build on second-order MTT because experiencing regret or relief requires one to realize that in the past (level 1) different options of the relative future (level 2) were available [48]. By comparing the actual present with an alternative present, the actual state of the world is evaluated as better (relief) or worse (regret) than the counterfactual alternative. Adding yet another layer of recursion, the anticipation of experiencing counterfactual emotions constitutes third-order MTT, which seems to emerge later in development than merely experiencing relief and regret themselves. Only from around age 8 or later do children report such anticipated regret when imagining that they will learn in the future (level 1) that—in the relative past (level 2)—better options of the relative future (level 3) were available [42,44,49,50]. While this might sound complicated, we engage in such reasoning quite naturally in our daily life. For instance, when contemplating whether to get a tattoo, we might anticipate that, in the future, we may regret our choice.

Against this background, our second research question is thus whether subsequent development in both MTT and ToM rely on the capacity to recursively embed representations and whether they therefore develop in a parallel and correlated manner [19,22]. To address the two research questions about the joint underlying foundation of the emergence and subsequent development of ToM and MTT, we compared children’s performance in first- to third-order ToM and MTT tasks in Study 1. In three follow-up studies, we then compared first-order ToM and MTT (Study 2a), second-order ToM and MTT (Study 2b) and third-order ToM and MTT (Study 2c) separately.

## 2. Study 1

The aim of Study 1 was to compare the emergence of ToM and MTT as well as their subsequent higher-order development within a single study across an age range (ages 3 to 8) that spans the emergence of ToM and MTT during early childhood and extends until middle school, where higher-order forms of both abilities would be expected to have developed.

### 2.1. Method

Preregistrations and supplementary materials for all four studies, including details on the samples, exclusion criteria, task protocols, materials, counterbalancing, data and analyses can be found on OSF (https://osf.io/8gv3t/).

#### 2.1.1. Design

This study was preregistered on AsPredicted (67697). Each child was tested in six tasks (see table 1) by one of three female experimenters in two separate test sessions. Children received three ToM tasks in test session A and three MTT tasks in test session B. The order of sessions was counterbalanced. All children participated remotely via a video conference (following the procedure by [51]).

**Table 1.** Conceptualization and operationalization of first-, second- and third-order Theory of Mind and Mental Time Travel tasks.

order	Theory of Mind		**Mental Time Travel**	
1	first-order false belief	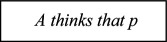	future possibilities	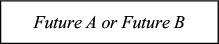
2	second-order false belief	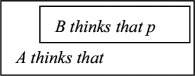	counterfactual reasoning	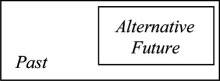
3	third-order false belief	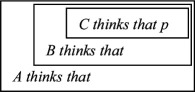	anticipation of regret	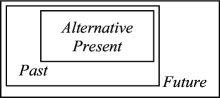

#### 2.1.2. Participants

One hundred and forty children between ages 3 and 8 (36–107 months) participated in the study. Twenty children had to be excluded from the analyses; thus, the final sample comprised 120 children (61 female, 59 male; 20 children per age group).

#### 2.1.3. First-order Mental Time Travel task

**Figure 1.**
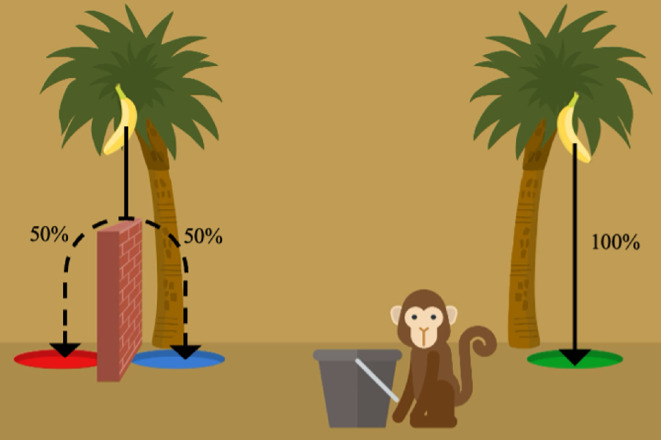
Screenshot of first-order MTT task with arrows indicating the possible trajectories of the falling bananas.

The task tested children’s ability to reason about future possibilities (adapted from [13]). After a familiarization procedure with several control questions, children were asked to help a monkey catch a banana in two test trials (‘In which hole should the monkey put the bucket, so that he catches a banana for sure?’; see figure 1). Children answered by naming the colour of the hole. Their answer was rated as correct if children chose the certain hole (e.g. in figure 1: the green hole).

#### 2.1.4. Second-order Mental Time Travel task

The task (adapted from [35]) tested children’s counterfactual reasoning abilities in two test trials. After a familiarization procedure with several control questions, children saw animated videos of two snowballs running down two opposite hills breaking a snowman that stood in the valley between them. In one test trial, children were asked a subtractive counterfactual test question (‘If the white snowball had not rolled down the hill this time, would the snowman then have broken?’). In the other test trial, children were asked an additive counterfactual test question (‘If this time the tree had been standing there on the steep hill (*animated finger pointed at the steep hill*), would the snowman then have broken?’). The order of trials was counterbalanced. Children’s answer was rated as correct if they affirmed the question.

#### 2.1.5. Third-order Mental Time Travel task

**Figure 2.**
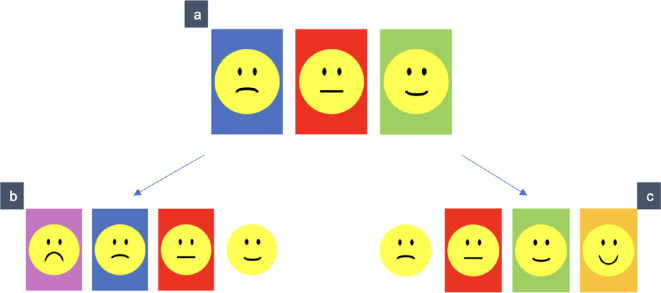
Smiley scale used in third-order MTT task. Example: if children answered the baseline question (a) with ‘happy’, the green smiley became the anchor for the scale shown during the test question (c). If children answered with ‘sad’, the blue smiley became the anchor (b). If children answered with ‘neither happy and nor sad’, the scale remained the same (a).

The task tested children’s ability to anticipate counterfactual emotions (adapted from [42]), specifically whether children would feel regret about a choice if they learned that there had been a better option in the past. After a familiarization procedure with several control questions, children received one control trial and one test trial. In both trials, children were asked to choose one of two boxes. Children always won one coin from their chosen box while the other box remained closed. After winning the coin, children gave a baseline rating of their emotion on a three-point smiley scale (‘How do you feel now that there was one coin in your box?’; see figure 2a). The corresponding smiley became the anchor of the scale for the subsequent test question, which differed between the control and the test trial (‘This is how you felt, when one coin was in your box. Imagine, that there was one coin [control trial]/five coins [test trial] in the other box, and you did not win it/them. How would you now feel about winning this box with one coin? Sadder, happier or the same?’ (with ‘this box’ referring to the box they chose; see figure 2b,c)). Children’s emotion ratings were then transformed to scores. Next, we computed a difference score between children’s emotion rating in the baseline and the test question for the control trial and the test trial. Children were classified as being able to anticipate regret if their emotional change in the test trial was more negative than in the control trial.

#### 2.1.6. Theory of Mind tasks

Children saw three animated video storylines that tested for their first-order false belief understanding (two test questions), second-order false belief understanding (two test questions) and third-order false belief understanding (one test question). The storylines that were used to measure first-order false belief understanding followed the standard change-of-location false belief task [52]: the child saw that Protagonist A placed his object in one of two boxes. Protagonist A then left and Protagonist B entered the scene, relocated the object to the other box and left again. After two control questions, the test question was asked: ‘When A comes back, where will A look for his object first?’. In the second video, this storyline was extended to assess second-order false belief understanding. In the third video, second- and third-order false belief understanding were measured with a story about a school soccer team in which children needed to track the players’ attitudes about each other’s mental states (adapted from [31]). Children first watched the video and then answered the following questions:

- Second-order false belief test question: ‘Which sentence is true?

(A) Max does not know that the soccer coach wants Max and Paul to both play on the soccer team. [correct answer]
(B) Max knows that the soccer coach wants Max and Paul to both play on the soccer team.’

- Third-order false belief test question: ‘Which sentence is true?

(A) The soccer coach thinks that Max believes that he wants Max to play on the soccer team.
(B) The soccer coach thinks that Max believes that he does not want Max to play on the soccer team.^1^ [correct answer]’

### 2.2. Results

#### 2.2.1. Coding

**Figure 3.**
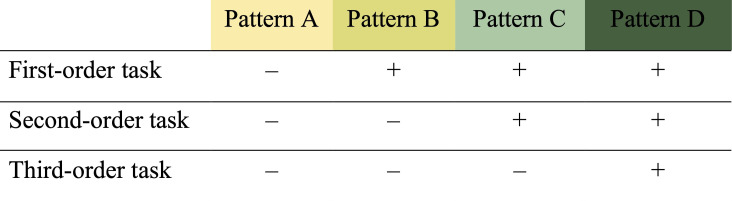
Performance patterns of success (+) and failure (–). Performance that deviated from these patterns was categorized as‘other’.

Correct trials were coded with ‘1’, incorrect trials with ‘0’. All trials of a task were added to a sum score. In the first- and second-order tasks, children received two test trials per task and were thus able to receive a sum score between 0 and 2. In the third-order tasks, children received one test trial and were thus able to receive a sum score between 0 and 1. To be categorized as passers (indicated by a ‘+’ in figure 3), children needed to solve all trials of a task correctly (i.e. two trials in the first- and second-order tasks and one trial in the third-order tasks). Children’s performance across the three tasks of one ability (ToM or MTT) was categorized in performance patterns. We expected children to perform according to one of four performance patterns (see figure 3). Performance that deviated from these patterns was categorized as ‘other’.

#### 2.2.2. Plan of analysis

All analyses were conducted using R 4.3.3 [53]. In a first step, we aimed to test whether the development of both ToM and MTT abilities follows a stepwise order. To ensure that this sequence indicates a progressive development, we ran separate Guttman scaling analyses on ToM and MTT [54]. To analyse the reliability of the ToM and MTT Guttman scales, coefficients of reproducibility and coefficients of scalability were computed. In a second step, we compared whether children’s performance pattern in ToM aligned with their performance pattern in MTT. To test the consistency of performance, we computed a Weighted Kappa of the cross table of the four expected performance patterns. Additionally, partial correlations of the sum scores between first-order, second-order and third-order tasks were calculated while controlling for children’s age in months.

#### 2.2.3. Development of Theory of Mind and Mental Time Travel

**Figure 4.**
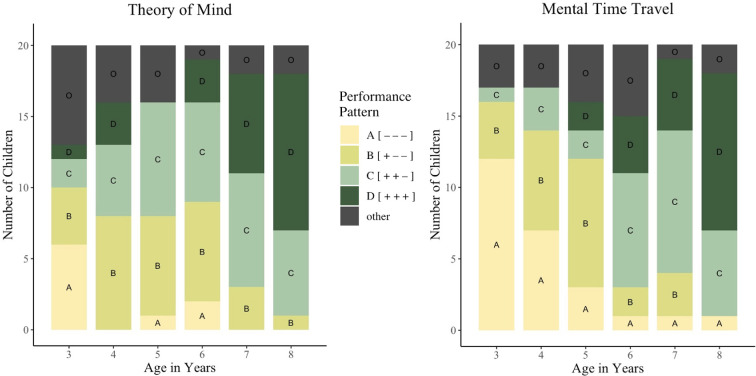
Number of children in each performance pattern as a function of their age in Theory of Mind (left) and Mental Time Travel tasks (right).

The proportion of children who passed each task is presented in table 2 for MTT tasks and in table 3 for ToM tasks. In general, performance in both ToM and MTT followed a stepwise order (see figure 4). Children first passed first-order tasks, then second- and finally third-order tasks. Performance of 83% of the children (100 of 120) fitted this three task Guttman scale of ToM tasks, and performance of 85% of the children (102 of 120) fitted the Guttman scale of MTT tasks. The coefficient of reproducibility [55] for the scalogram analysis of ToM tasks was *C_R_(ToM*) = 0.89 and *C_R_(MTT*) = 0.90 for MTT tasks [56]. The coefficient of scalability is *C_S_(ToM*) = 0.66 for ToM, and *C_S_(MTT) =* 0.72 for MTT [57].

**Table 2.** Percentage of children passing Mental Time Travel tasks across age groups.

	3-year-olds	4-year-olds	5-year-olds	6-year-olds	7-year-olds	8-year-olds
MTT 1	30	50	70	75	90	95
MTT 2	15	20	25	70	80	85
MTT 3	5	10	25	35	25	65

**Table 3.** Percentage of children passing Theory of Mind tasks across age groups.

	3-year-olds	4-year-olds	5-year-olds	6-year-olds	7-year-olds	8-year-olds
ToM 1	35	95	85	85	95	95
ToM 2	25	45	40	55	80	90
ToM 3	35	30	20	20	40	65

#### 2.2.4. Consistency of performance across Theory of Mind and Mental Time Travel

**Figure 5.**
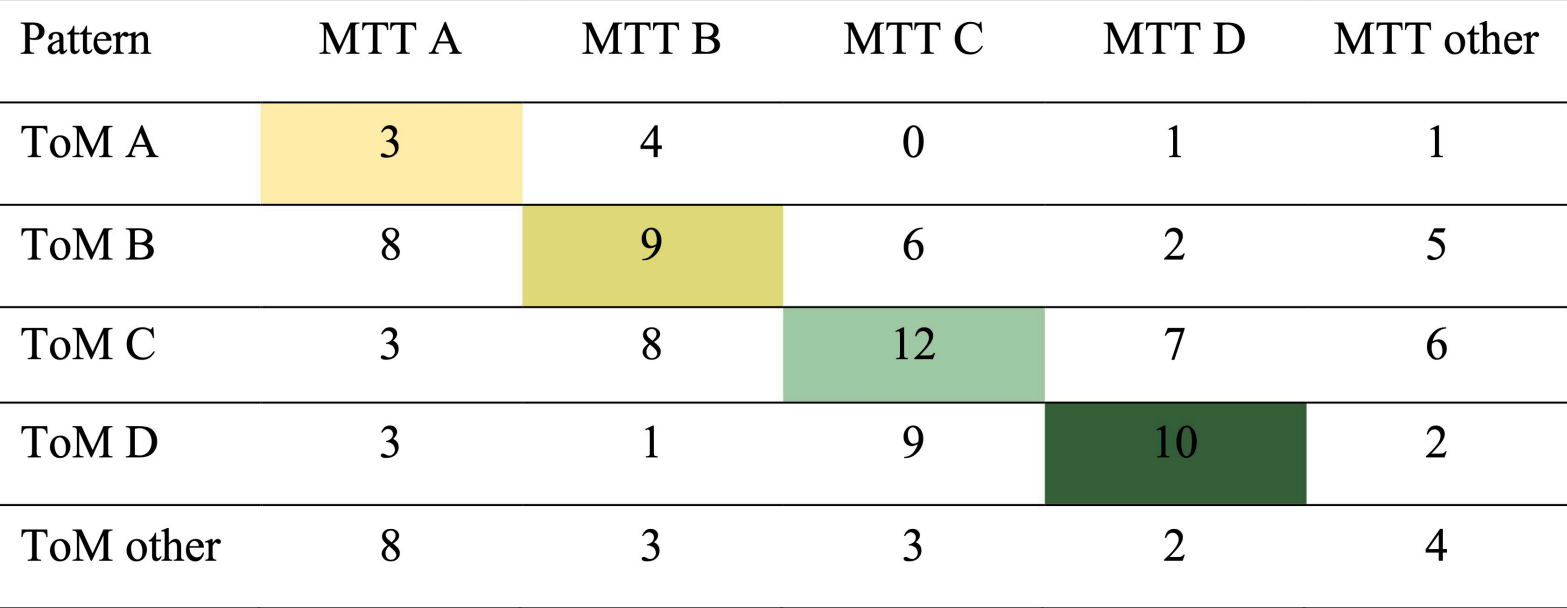
Number of children per performance pattern in Theory of Mind and Mental Time Travel tasks. Pattern A reflects failure across all tasks, pattern B reflects success only in the first-order task, pattern C reflects success in first- and second-order tasks, pattern Dreflects success across all tasks.

Figure 5 shows children’s performance patterns in MTT as a function of their performance pattern in ToM. The consistency of performance (without category ‘other’) is moderate (*K* = 0.31). Partial correlations between the two first-, second- and third-order tasks respectively showed significant results only for the second-order tasks (*r* = 0.26, *p* = 0.005), but not for the first-order (*r* = −0.03, *p* = 0.747) and the third-order tasks (*r* = 0.11, *p* = 0.229) when controlling for children’s age in months.

### 2.3. Discussion

Study 1 investigated whether ToM and MTT build on joint underlying cognitive foundations and thus emerge and develop in a parallel and correlated manner. To this end, Study 1 compared children’s performance in first- to third-order ToM and MTT tasks across an age range that encompassed the emergence of first forms of both abilities as well as the development of higher-order forms. The results of Study 1 show a stepwise development of orders for both abilities separately. This is reflected in children’s performance patterns that indicate a progressive development (see figure 4) such that, overall, children first pass first-order, then second-order and finally third-order tasks. However, Study 1 found only mixed evidence for a potential joint foundation and subsequent parallel development of the two abilities: while the consistency of performance between ToM and MTT was moderate, only the correlation between second-order false belief reasoning and counterfactual reasoning turned out to be of significance. Therefore, Study 1 does not provide stringent correlational evidence for a joint cognitive foundation of ToM and MTT and their subsequent higher-order development. However, since Study 1 tested all children in all tasks of one ability within a single test session, we could only administer a limited amount of test trials per order. Study 1 might thus not have had enough test power to reveal robust correlations between ToM and MTT. We therefore decided to run three separate follow-up studies (figure 2a–c), one for each order of recursion, to increase the test power by doubling the amount of test trials in the MTT tasks. Each follow-up study focused on one order of recursion, measured in both ToM and MTT. The age range of each follow-up study was narrower and targeted around the age of emergence of the order of recursion under study.

## 3. Study 2a

Study 2a investigated the emergence of first-order ToM (first-order false belief reasoning) and first-order MTT (reasoning about future possibilities). We narrowed the age range to 3 to 4 years, as this is typically the age were first-order false belief understanding (e.g. [18]), and the ability to reason about future possibilities (e.g. [17,19]), start to emerge, which was further corroborated by the results of Study 1.

### 3.1 Method

#### 3.1.1. Design

This study was preregistered on AsPredicted (107717). Each child received two first-order ToM test trials and four first-order MTT test trials. ToM and MTT trials were presented in blocks, whose order was counterbalanced. Children were tested in one test session by one of two experimenters in the laboratory.

#### 3.1.2. Participants

One hundred and one children between ages 3 and 4 (36–58 months) participated in the study. Nine children had to be excluded from the analyses; thus, the final sample comprised 92 children^2^ (46 female, 46 male; 46 children per age group).

#### 3.1.3. Materials and procedure

Both first-order MTT and ToM tasks followed the same logic as in Study 1 but were adapted for laboratory testing. In the first-order MTT task, children saw two transparent tubes attached to a wooden board. One of the tubes was shaped like an inverted Y with one entrance and two exits, while the other tube had only one branch with one exit. After a demonstration phase, children received four test trials, where they had to help a monkey catch an object. Children prepared to catch the object by pushing a toy truck under a tube exit (e.g. ‘These two red stones will fall through the tubes. You need one red stone. Look closely where a red stone is sure to come out! Where do you place the truck?’). Children’s answer was rated as correct when they placed the wagon under the tube with only one exit. In the first-order ToM task, the same change-of-location storyline as in Study 1 was narrated by the experimenter and enacted with cardboard boxes and figurines.

### 3.2. Results and discussion

#### 3.2.1. Coding

Correct trials were coded with ‘1’, incorrect trials with ‘0’. All trials of a task were added to a sum score. Children were able to receive a sum score between 0 and 4 in the first-order MTT task and between 0 and 2 in the first-order ToM task.

#### 3.2.2. Performance in first-order Theory of Mind and Mental Time Travel tasks

The descriptive results are displayed in table 4.

**Table 4.** Percentage of correct responses in the test trials of the first-order Theory of Mind and Mental Time Travel tasks.

	3-year-olds	4-year-olds
first-order ToM	51.06	75.56
first-order MTT	52.13	61.67

#### 3.2.3. Relationship between first-order Theory of Mind task and first-order Mental Time Travel task

The correlation between the sum scores of the first-order ToM and first-order MTT task controlling for children’s age in months showed no significant relationship between the tasks (*r* = 0.13, *p* = 0.204).^3^ The results of additional preregistered analyses can be found on OSF. Taken together, Study 2a did not find a correlation between the first-order ToM and MTT tasks and does therefore not provide evidence for a joint cognitive foundation of the two abilities.

## 4. Study 2b

Study 2b investigated the development of second-order ToM (second-order false belief reasoning) and second-order MTT (counterfactual reasoning). We narrowed the age range to 4.5 to 7.5 years, as this spans the age at which the understanding of second-order false beliefs [30] and the ability to reason counterfactually about physically overdetermined events (e.g. [35]) emerge, which was further corroborated by the results of Study 1.

### 4.1. Method

#### 4.1.1. Design

This study was preregistered on AsPredicted (101410). Each child received two second-order ToM test trials and four second-order MTT test trials. The tasks were presented alternately (e.g. ToM, MTT, ToM, MTT). The order of tasks and test questions was counterbalanced. Children were tested in one test session in a non-moderated online study that was programmed and conducted using *Labvanced*. The families received a link via email and used a laptop or desktop computer to complete the study at home.

#### 4.1.2. Participants

One hundred and eight children between age 4.5 and 7.5 (54–88 months) participated in the study. Eighteen children had to be excluded from the analyses; thus, the final sample comprised 90 children (45 female, 45 male; thirty 4.5- to 5.5-year-olds, thirty 5.5- to 6.5-year-olds and thirty 6.5- to 7.5-year-olds).

#### 4.1.3. Materials and procedure

The material for both tasks was identical to Study 1, the only differences being that the control and test questions were read out by a prerecorded voice and that a second MTT storyline was added. Children or their caretakers were required to click on buttons or on the respective boxes on the screen to provide their answers.

### 4.2. Results and discussion

#### 4.2.1. Coding

Correct trials were coded with ‘1’, incorrect trials with ‘0’. All trials of a task were added to a sum score. Children were able to receive a sum score between 0 and 4 in the second-order MTT task and between 0 and 2 in the second-order ToM task.

#### 4.2.2. Descriptive results

The descriptive results are displayed in table 5.

**Table 5.** Percentage of correct responses in the test trials of the second-order Theory of Mind and Mental Time Travel tasks.

	4.5- to 5.5-year-olds	5.5- to 6.5-year-olds	6.5- to 7.5-year-olds
second-order ToM	51.67	63.79	82.26
second-order MTT overall	22.50	51.72	73.39
additive	8.33	48.28	69.35
subtractive	36.67	55.17	77.42

#### 4.2.3. Relationship between second-order Theory of Mind and second-order Mental Time Travel

The correlation between the sum scores of the second-order ToM and second-order MTT task controlling for children’s age in months showed no significant relationship between the tasks (*r* = 0.17, *p* = 0.104). The results of additional preregistered analyses can be found on OSF. While Study 1 found a correlation between second-order ToM and MTT, Study 2b did not replicate that finding. Since the amount of MTT test trials was doubled in Study 2b, it is unlikely that we did not replicate the finding due to insufficient test power. Study 2b does therefore not provide evidence for a parallel and coordinated higher-order development of ToM and MTT that is based on recursively embedding representations.

## 5. Study 2c

Study 2c investigated the development of third-order ToM (third-order false belief reasoning) and MTT (anticipation of counterfactual emotions). We chose the age range of 7 to 9 years, as this spans the age at which the understanding of third-order false beliefs [30] and the ability to anticipate counterfactual emotions (e.g. [42,49]) emerge. However, we expanded the age range to include 9-year-olds as the 8-year-old children in Study 1 were still far from ceiling performance.

### 5.1. Methods

#### 5.1.1. Design

This study was preregistered on OSF (https://osf.io/kgu8e). Each child received two third-order ToM test trials and two third-order MTT test trials. The tasks were presented alternately (e.g. ToM, MTT, ToM, MTT). Children were tested in one test session by one of two experimenters in the laboratory or at a childcare programme during the school holidays. The tasks were presented on a laptop screen.

#### 5.1.2. Participants

One hundred children between age 7 and 9 (84–119 months) participated in the study. Seven children had to be excluded from the analysis; thus, the final sample comprised 93 children^4^ (49 female, 44 male; 31 children per age group).

#### 5.1.3. Materials and procedure

Both third-order ToM and MTT tasks followed the same logic as in Study 1. However, the procedure of the third-order MTT task differed in the following ways: firstly, since the study was in-person, children now collected coins in a treasure chest that they could swap for a present after the study. Secondly, before children rated their emotion after winning one coin (baseline rating), the other box was revealed to be empty. Thirdly, we increased the number of coins in the counterfactual scenario from 5 to 10. Fourthly, we did not use a control trial and the smiley scale to measure children’s change of emotion. Instead, in each test trial, children were first asked how they felt about having chosen the box, where they had won one coin (e.g. most children answered with ‘good’). Then, children were asked the test question (e.g. ‘Imagine that the [other] orange box was not empty but that there were ten coins in the orange box. But you have only chosen the green box. Would you still find that as [what child said, e.g. ‘good’] as before?’). Children were classified as being able to anticipate regret if their answer indicated that they would like their choice less if there were 10 coins in the other box.

### 5.2. Results and discussion

#### 5.2.1. Coding

Correct trials were coded with ‘1’, incorrect trials with ‘0’. All trials of a task were added to a sum score. Children were able to receive a sum score between 0 and 2 in each task.

#### 5.2.2. Descriptive results

The descriptive results are displayed in table 6.

**Table 6.** Percentage of correct responses in the test trials of the third-order Theory of Mind and Mental Time Travel tasks.

	7-year-olds	8-year-olds	9-year-olds
third-order ToM	58.06	74.19	72.58
third-order MTT	54.84	53.23	67.74

#### 5.2.3. Relationship between third-order Theory of Mind and third-order Mental Time Travel

The correlation between the sum scores of the third-order ToM and third-order MTT task controlling for children’s age in months showed no significant relationship between the tasks (*r* = –0.002, *p* = 0.982). The results of additional preregistered analyses can be found on OSF. Taken together, Study 2c did not find a correlation between the third-order ToM and MTT and does therefore not provide evidence for a parallel and coordinated higher-order development of ToM and MTT on the third order of recursion.

## 6. General discussion

The guiding questions of the present study were whether two cognitive abilities that enable us to go beyond representing the here and now, namely ToM and MTT, build on some form of joint cognitive foundation and thus emerge and develop in parallel and correlated ways across childhood. It has been suggested that, for example, capacities for simulation and projection or for meta-representation may lie at the core of both abilities and that more complex, higher-order forms may emerge by recursive embedding [19,22]. To address these questions, we compared children’s performance in first- to third-order ToM and MTT tasks across four studies to delineate the emergence of both abilities and their subsequent development. To our knowledge, this study was the first attempt to directly compare children’s developing abilities in ToM and MTT across different levels of recursion.

Overall, evidence from earlier studies investigating both abilities separately was confirmed: we found parallel developmental trajectories in the two domains. The results of Study 1 show a stepwise development of passing first-, second- and third-order tasks in both ToM and MTT separately and a moderately consistent performance pattern across both abilities. However, our studies do not provide conclusive evidence for actual associations of the two abilities. Study 1 only found a relation between second-order false belief reasoning and counterfactual reasoning; however, this finding could not be replicated in Study 2b. Thus, across four studies, we did not find robust correlations between the two abilities on any order of recursion and, consequently, no stringent evidence for a joint cognitive foundation.

Nevertheless, while we did not find clear evidence for a relation of ToM and MTT in our studies, this absence of evidence does not necessarily amount to the evidence of absence of such a relation. There might be a relationship between children’s developing abilities in the two domains that could not be reliably shown in the present studies for various reasons about which we can only speculate. For instance, we may have failed to detect a relationship due to the methodological implementation of the tasks, specifically the operationalization of children’s MTT abilities. The three orders of MTT were operationalized as reasoning about future possibilities, counterfactual reasoning about past events and the anticipation of counterfactual emotions. We decided to use established tasks from the literature that measure these abilities in children. Consequently, these tasks had not been developed to mirror the structure of ToM tasks. We may have thus failed to detect a relationship, as the ToM and MTT tasks were not matched closely enough (see [58] for an example of a closely matched false belief and counterfactual reasoning task).

Moreover, our implementations of the MTT tasks might have been artificially difficult as they imposed task demands that went beyond MTT. For instance, the first-order MTT tasks may have posed additional task demands in terms of intuitive physics. In Study 1 and Study 2a, children were asked to catch one of two objects that were either falling from trees or through transparent tubes. Children’s performance in these tasks might depend on their ability to simulate the trajectories of the falling objects in addition to their MTT abilities. First support for the relevance of the operationalization in possibility reasoning tasks comes from a study that required reasoning about future possibilities based on object identities rather than physical trajectories [59]. In this task, even 3-year-olds performed competently, thus, performance diverged strikingly from studies that used tasks relying on physical trajectories (e.g. [13–16]). In addition, the first-order MTT task in the current study may not only have measured the ability to reason about future possibilities but also logical reasoning abilities (e.g. understanding of disjunction and exclusion), which may have made our task artificially harder.

Another reason why the present studies may have failed to detect a relationship between ToM and MTT is the higher probability of false positives in the ToM tasks. For one, children reason between limited alternatives in these tasks: if a child does not know the answer to the third-order ToM test question and simply guesses, the probability of guessing correctly is 50%—this is a lot higher than the probability of randomly displaying the correct response pattern in the third-order MTT task. Moreover, it has been argued that, given these limited answer alternatives, children can sometimes solve higher-order ToM tasks through shorter recursive chains [60], although the current study tried to limit this possibility as much as possible. Moreover, another reason why we failed to find correlations between the third-order tasks may be that the children did not deploy the intended level of recursively embedded representations in the third-order MTT tasks. For instance, in Study 2c, children may have simply solved the task by deploying second-order MTT (‘How would I feel now if the other box had been revealed to contain a different amount of coins in the past?’).^5^

Another possibility is that we failed to find evidence for a joint meta-representational core, as the proficiency and flexibility with which children engage in meta-representational thought might vary with its content. The amount of experience in the two domains might diverge as children may be more familiar with mental state reasoning than with MTT. Children’s life is full of social interactions with their parents, siblings, peers and teachers, which may actively motivate children to engage in mental state reasoning. Moreover, training studies show that children’s performance in ToM tasks benefits from practice in mental state reasoning [61]. By contrast, reasoning about other points in time might be less relevant or salient in children’s daily life. Children may not be as frequently confronted with situations that require them, for instance, to explicitly reason counterfactually. However, our argument is not about the daily relevance of an ability *per se,* but rather how transparently or explicitly the recursive structure is expressed in language. In Theory of Mind, recursion is more evident in linguistic expression (e.g. ‘She thinks that he believes that she knows…’). By contrast, in MTT, higher-order recursive structures are rarely reflected in the surface structure of language. Future studies could compare children’s performance in less familiar meta-representational task implementations with their emerging MTT abilities to rule out possible training effects.

## 7. Conclusion and outlook

To our knowledge, the present study was the first to directly compare the development of the cognitive abilities ToM and MTT from their first emergence in early childhood to the acquisition of higher-order forms during middle childhood. We found that the developmental trajectories of both abilities appear to run in parallel and progress in a stepwise order, which indicates a recursive progression. Nevertheless, beyond these parallel trajectories, the present studies did not find robust correlations between ToM and MTT at the various levels and thus do not provide stringent evidence for a joint underlying cognitive foundation of the two capacities—be it meta-representational thought or simulation and projection. This absence of evidence does not necessarily amount to evidence of absence, however. To further explore the possibility of a relation between the two abilities, future studies could systematically address the limitations of the present studies by trying to match ToM and MTT tests more closely in terms of their structure and the task demands.

## Data Availability

Preregistrations and electronic supplementary material for all four studies, including details on the samples, exclusion criteria, task protocols, materials, counterbalancing, data and analyses, can be found on OSF [62].

## Footnotes

^1^
In German, all sentences were formed with ‘want that’-complement (‘möchte, dass’).

^2^
We pre-registered to test until we can include 90 children. However, due to a scheduling error two additional children participated in the study and were included in the analyses.

^3^
We would like to acknowledge a *post hoc* analysis conducted by one of the reviewers, which examined whether the strength of the correlation between tasks varied across the four MTT trials. This analysis revealed a strong partial correlation, controlling for age (*r* = 0.541, *p* < 0.001), between performance on trials 3–4 of the first-order MTT task and the first-order ToM task among 3-year-olds, but not among 4-year-olds. No significant correlations were found between trials 1–2 of the first-order MTT task and the first-order ToM task in either age group.

^4^
We pre-registered to test until we can include 90 children. However, due to a scheduling error three additional children participated in the study and were included in the analyses.

^5^
We thank Jonathan Redshaw for raising this point.
